# Employing the therapeutic operating characteristic (TOC) graph for individualised dose prescription

**DOI:** 10.1186/1748-717X-8-55

**Published:** 2013-03-07

**Authors:** Aswin L Hoffmann, Henk Huizenga, Johannes HAM Kaanders

**Affiliations:** 1 Department of Radiation Oncology, Radboud University Nijmegen Medical Center, P.O. Box 9101, Nijmegen, 6500 HB, The Netherlands; 2 Department of Radiation Oncology (MAASTRO), GROW School for Oncology and Developmental Biology, Maastricht University Medical Center, P.O. Box 1588, Maastricht, 6201 BN, The Netherlands

**Keywords:** Radiotherapy, Treatment planning, Individualisation, Dose-response relations, Decision-making

## Abstract

**Background:**

In current practice, patients scheduled for radiotherapy are treated according to ‘rigid’ protocols with predefined dose prescriptions that do not consider risk-taking preferences of individuals. The therapeutic operating characteristic (TOC) graph is applied as a decision-aid to assess the trade-off between treatment benefit and morbidity to facilitate dose prescription customisation.

**Methods:**

Historical dose-response data from prostate cancer patient cohorts treated with 3D-conformal radiotherapy is used to construct TOC graphs. Next, intensity-modulated (IMRT) plans are generated by optimisation based on dosimetric criteria and dose-response relationships. TOC graphs are constructed for dose-scaling of the optimised IMRT plan and individualised dose prescription. The area under the TOC curve (AUC) is estimated to measure the therapeutic power of these plans.

**Results:**

On a continuous scale, the TOC graph directly visualises treatment benefit and morbidity risk of physicians’ or patients’ choices for dose (de-)escalation. The trade-off between these probabilities facilitates the selection of an individualised dose prescription. TOC graphs show broader therapeutic window and higher AUCs with increasing target dose heterogeneity.

**Conclusions:**

The TOC graph gives patients and physicians access to a decision-aid and read-out of the trade-off between treatment benefit and morbidity risks for individualised dose prescription customisation over a continuous range of dose levels.

## Background

The main task in radiation dose prescription and treatment planning is to maximise the tumour control probability (TCP) while maintaining an acceptable normal tissue complication probability (NTCP). Currently, this compromise is ‘frozen’ in treatment protocols, which are based on consensus opinions about what is considered to be the best trade-off for a specific patient population. However, in an era where patient empowerment enters into clinical practice, subjective criteria reflecting the physician’s or individual patient’s risk-taking preferences should inherently be taken into account to establish a ‘customised’ treatment. Therefore, individualised treatment prescription and planning requires decision-making based on TCP and NTCP scores rather than on dosimetric criteria alone.

Amols *et al.* proposed an *a posteriori* decision-making approach to rank existing treatment plans having different combinations of TCP and NTCP based on a single figure of merit quantifying the physician’s preferences [1]. Another approach is to extract patient preferences prior to treatment. In a recent prospective trial involving patients with localised prostate carcinoma scheduled for three-dimensional conformal radiotherapy (3D-CRT), patients were offered an *a priori* choice between treatments with two alternative dose levels resulting in different probabilities for tumour control and side-effects [2,3]. From this study, it became clear that the majority (79%) of patients opted to be involved in the choice of their treatment once a decision-aid was provided, and most patients attached a higher weight to quality-of-life related aspects than to tumour control [4].

The availability of modern treatment techniques provides the ability to design a variety of treatment plans with divergent trade-offs between benefit and morbidity. The ultimate way to take this variety into account is to predict TCP and NTCP scores for the individual patient based on the 3D dose distribution and fractionation scheme applied. This is challenging, since the clinical outcome measures for these treatments are not well known. Furthermore, specific tumour characteristics determining radiation response (*e.g.*, intrinsic radiosensitivity and hypoxia) are often unknown for the individual patient. Nevertheless, various models and parameter sets to estimate TCP and NTCP from a 3D dose distribution have been developed and applied (*e.g.*, [5-8]). It is generally believed that these models are adequate to rank rival treatment plans provided they are used over the dose range from which they have been derived. Some of these models have been incorporated into modern treatment planning systems, enabling a ‘radiobiological’ evaluation of treatment plans. However, the TCP/NTCP trade-off is difficult to assess in current treatment planning systems, even for a simple ‘dose scalarisation’ approach where only the prescription dose of a given treatment plan is changed for either a fixed number of fractions or for a fixed fraction dose. The concept of maximizing the probability of uncomplicated tumour control, *P*_+_, as a function of dose has been proposed to find the single optimum dose level for this approach [9,10]. The criticism against this measure is that an *a priori* ‘rigid’ trade-off between TCP and NTCP is assumed without knowing their interrelationship over the range of potential dose prescriptions. As such, it is not suitable as a single measure for dose prescription customisation.

The aim of the present paper is to apply the concept of the therapeutic operating characteristic (TOC) graph to assess the trade-off in the TCP/NTCP domain over a continuous range of prescribed dose levels for given treatment plans. The TOC graph is presented as an interactive tool for dose prescription customisation of a treatment plan for an individual patient. We compare the *P*_+_ and TOC graph and discuss their value for individualised dose prescription optimisation. The concept is illustrated by a clinical example of prostate cancer where the trade-off between 5-year biochemical no evidence of disease (bNED_5_), late gastrointestinal (GI) and genitourinary (GU) morbidity is studied.

## Methods

### Therapeutic operating characteristic (TOC) graph

The TOC is a parametric plot of TCP *vs.* NTCP with the prescribed dose as a continuous independent parameter [11-15]. As TCP and NTCP increase with total dose, their interrelationship presents an ascending curve in the benefit-injury decision space. The TOC graph can be used to estimate the optimal level of therapeutic effect and the associated dose level, based on individual risk-taking preferences.

So far, few quantitative measures of therapeutic window or therapeutic power have been published. Both refer to a quality index for radiotherapy to achieve loco-regional tumour control and to prevent severe late side effects. We propose to use the area under the curve (AUC) of the TOC graph as an index of the therapeutic power of a treatment technique or plan, independent of consensus on the prescribed dose level. This is by analogy with the AUC of the ROC graph used in diagnostic radiology [16].

### TOC graph for a patient population: modelling results from clinical studies

The TOC graph was first applied to clinical outcome data from a systematic literature review on the effects of radiation dose on tumour control and morbidity in the treatment of prostate cancer [3]. The trade-off between TCP (*i.e.*, bNED_5_), NTCP_GI_ and NTCP_GU_ (late GI and GU morbidity Grade ≥2 RTOG) was assessed for three-dimensional conformal radiotherapy (3D-CRT) techniques of combined data from dose escalation studies. The models and parameters used to describe the dose-effect relationship for the patient population studied are summarised in Additional file 1.

### TOC graph for an individual patient: technique assessment and dose prescription customisation

TCP and NTCP models derived from the literature were applied to a 3D dose distribution of a given initial treatment plan to construct TOC graphs by *a posteriori* variation of the total prescription dose. This was either accomplished by variation of the number of fractions (at constant dose-per-fraction assuming no tumour cell repopulation correction for overall treatment time) or by variation of the fraction dose (while keeping the number of fractions constant). TOC graphs were generated after treatment planning to allow for TCP/NTCP balancing and selection of the preferred prescription for therapy delivery. Taking the individual’s risk-taking preferences into account, a treatment plan with a customised dose prescription can be selected as a point on the TOC graph. We illustrate the concept of dose-level scaling for a typical prostate cancer patient using different treatment delivery and plan optimisation techniques in a step-wise approach. Firstly, TCP/NTCP evaluation was done for a forward planned 3D-CRT dose distribution that had initially been designed for standard fractionation. Secondly, a TOC graph was generated for an intensity-modulated radiotherapy (IMRT) plan obtained by inverse planning using dose and dose-volume (*i.e.*, physical) objectives with equivalent initial prescribed dose (IMRT_phys_), as it was expected that the NTCP of the IMRT_phys_ plan was lower under tumour iso-effective conditions than for the 3D-CRT plan. Then a third plan (IMRT_biol_) was obtained from the IMRT_phys_ plan by physico-biological optimisation, improving the TCP under isotoxic conditions. The hypothetical benefit of scaling the IMRT_biol_ was also assessed. TOC graphs and AUCs of the three plans were compared.

### Organ segmentation and standard treatment plans

Both the 3D-CRT and IMRT_phys_ plans had the same dose prescription recipe: 78 Gy in 39 fractions satisfying the 95% and 107% under- and overdosage criteria according to ICRU 50 criteria. The planning target volume (PTV) encompassed the prostate gland and base of the seminal vesicles plus a 5 mm isotropic margin. The rectum and bladder were delineated as organs at risk (OARs). The 3D-CRT plan comprised a wedged 10 MV co-planar 4-beam arrangement. The IMRT_phys_ plan encompassed a 10 MV co-planar 5-beam geometry and was generated for a maximum of 60 step-and-shoot segments by inverse treatment planning (Pinnacle^3^ version 7.6c; Philips Radiation Oncology Systems, Fitchburg, USA) with direct machine parameter optimisation (DMPO; RaySearch Laboratories AB, Stockholm, Sweden).

### Physico-biological treatment plan optimisation

For isotoxic optimisation, values for NTCP_GI_ and NTCP_GU_ were calculated from the IMRT_phys_ plan, and constituted upper limits for the IMRT_biol_ plan at fixed fraction number (*N* = 39). The physical objectives from the IMRT_phys_ plan were converted into constraints, resulting in the following physico-biological optimisation problem:

$$\begin{array}{ll} \text{maximise} & \text{TCP}\left(\underline{D}\right)\\ \text{subject}\;\text{to} & {\text{NTCP}}_{\text{GI}}\left(\underline{D}\right)\leq \text{ntc}p_{\text{GI}}\\ & {\text{NTCP}}_{\mathrm{GU}}\left(\underline{D}\right)\leq \text{ntc}p_{\text{GU}}\\ & {\text{DVH}}_{i}\left(D,V_{j}\right)\leq \text{dv}h_{i,j} \end{array}$$

where D is the dose distribution to be optimised, and *ntcp*_GI_, *ntcp*_GU_, *dvh*_i,j_ are constraint values for organ *i* and dose-volume constraint *j* as obtained from the IMRT_phys_ plan after it had been generated by inverse planning. Non-clinical research software (ORBIT Workstation, version 1.5; RaySearch Laboratories AB, Stockholm, Sweden) was used to solve this problem with DMPO [17]. The dose-response models used are summarised in Additional file 2.

For TOC analysis, treatment plans were retrieved from Pinnacle^3^ and ORBIT Workstation into an in-house developed software tool (MATLAB version 7.6.0; The MathWorks Inc., Natick, USA). For the tumour a conservative (α/β)_T_ = 2 Gy adopted from [18] was applied, while for both OAR endpoints generally accepted values of (α/β)_OAR_ = 3 Gy and 6 Gy were adopted for Grade ≥2 late GI [19] and late GU toxicity [20], respectively. These (α/β) ratios were used to account for voxel-based fractionation correction prior to calculating TCP and NTCP scores.

## Results

### TOC graph for patient population

**Figure 1:**
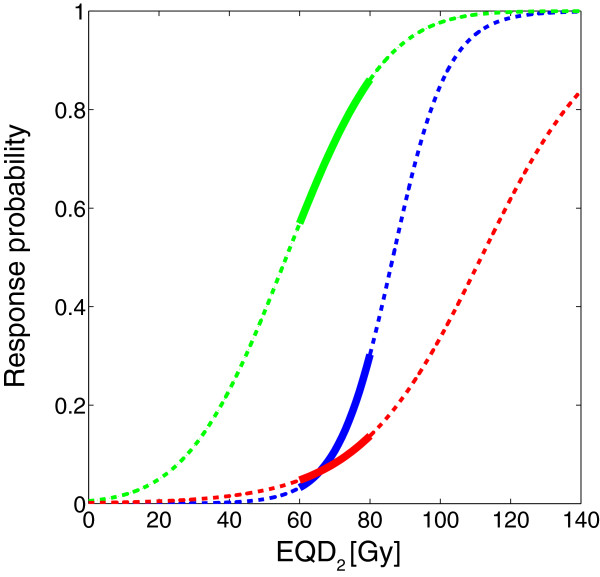
**Dose response graphs obtained from systematic literature review.** TCP (green), NTCP_GI_ (blue) and NTCP_GU_ (red) as a function of the prescribed total dose in 2 Gy fractions, obtained from a systematic literature review over the dose range of 60–80 Gy for 3D-CRT [3]. Dashed curves represent the relationship over extrapolated dose ranges.

Figure 1 illustrates the population-averaged dose-response graphs for TCP and NTCP as a function of the prescribed dose level in the 2 Gy equivalent dose (EQD_2_) range of 60–80 Gy, obtained from a systematic literature review [3].

**Figure 2:**
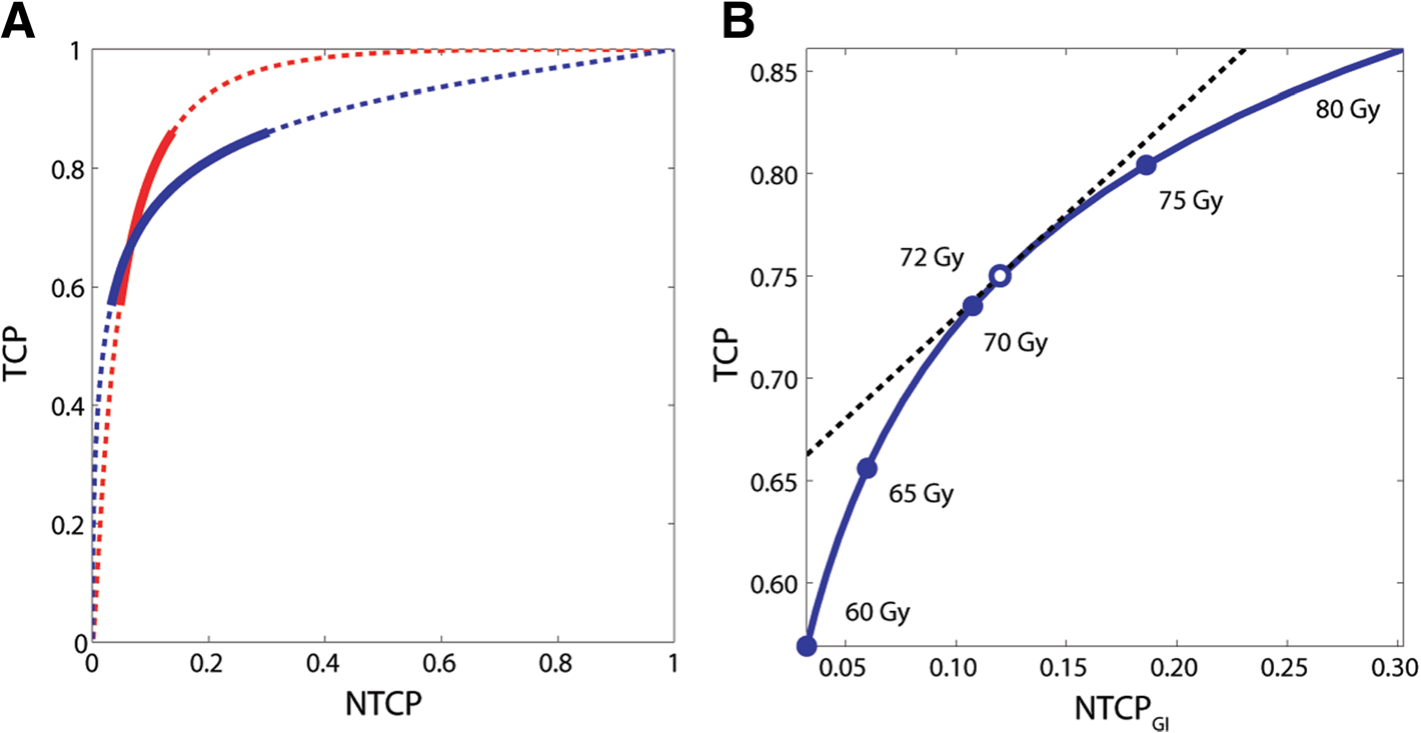
**TOC graphs reconstructed from systematic literature review.** TOC graphs showing (**A**) the trade-offs between TCP and either NTCP_GI_ (blue) or NTCP_GU_ (red) over the dose range of 60–80 Gy in 2 Gy fractions (solid curve) and the extrapolated dose ranges (dashed curve). In (**B**) the dashed line represents the tangent where TCP and NTCP_GI_ equally increase with dose and defines the optimum of *P*_*+*_ *= TCP - NTCP* (○). Dose levels in 2 Gy fractions are indicated (●) along the curve.

In Figure 2A, two TOC graphs depict the trade-offs between the TCP, NTCP_GI_, and NTCP_GU_ of Figure 1 when plotted against each other. Figure 2B illustrates the TOC graph zoomed in on the EQD_2_ range of 60–80 Gy for GI morbidity. In this graph, the dose level for which the increase in TCP and NTCP with dose is equal, and hence *P*_*+*_ *= TCP – NTCP* achieves its maximum value, is 72 Gy with associated TCP = 75% and NTCP = 12%. Below this level, the gain in TCP per unit dose is larger than the increase in NTCP, whereas the converse is true beyond this level.

**Figure 3:**
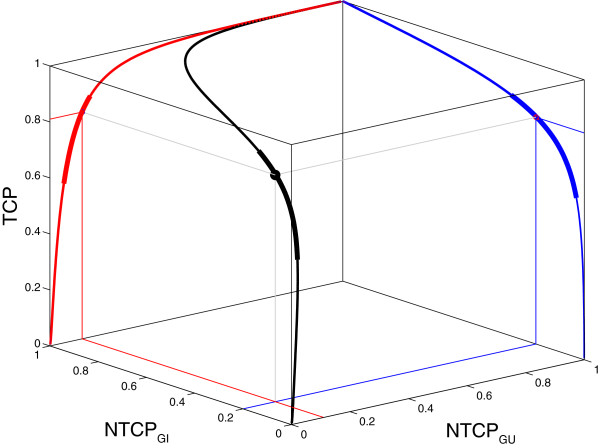
**3D TOC graph reconstructed from systematic literature review.** Multidimensional TOC graph (black) for TCP, NTCP_GI_, and NTCP_GU_ as a function of the prescribed dose in 2 Gy fractions. Projection of TOC graphs for TCP *vs.* NTCP_GU_ (red) and TCP *vs.* NTCP_GI_ (blue) are shown. Thin curves represent the relationship over extrapolated dose ranges; the 78 Gy plan is indicated (●).

In Figure 3, the interdependence between TCP, NTCP_GI_ and NTCP_GU_ as a function of the prescribed dose is shown in a 3D TOC graph together with its 2D projections on the TCP/NTCP_GI_ and TCP/NTCP_GU_ space that is shown in Figure 2A.

### TOC graphs for individual patient

**Figure 4:**
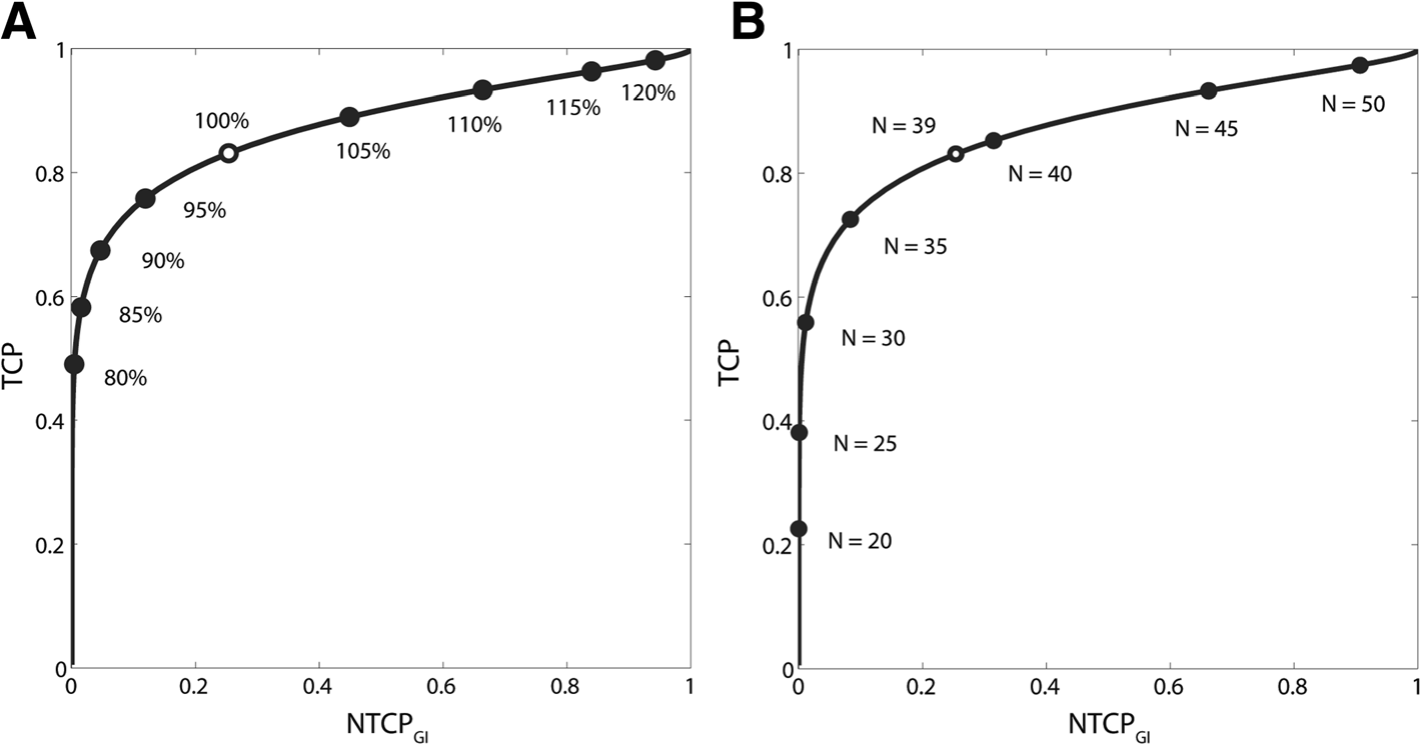
**TOC graphs for fraction size and fraction number variation.** TOC graphs for prostate 3D-CRT plan showing trade-off between TCP and NTCP_GI_ as a function of (**A**) the dose-per-fraction scale factor (at constant fraction number) and (**B**) the number of fractions *N* (at constant fraction dose). The initial plan (39 × 2 Gy) is marked (○) at the 100% dose level and at *N* = 39, respectively.

In Figures 4A and 4B, TOC graphs are shown for the initial (*i.e.*, 39 × 2 Gy) 3D-CRT treatment plan (TCP = 83%, NTCP = 25%) as a function of the scaled dose per fraction and the number of fractions, respectively. Dose statistics for renormalised plans are given in Table 1. It is evident that both curves coincide at the nominal dose prescription of 2 Gy per fraction or *N* = 39 fractions. Their relative position in the TCP/NTCP space is insensitive to the model parameters (TD_50_, γ_37_, *m*, *a)*, and only depends on the ratio of (α/β)_T_ to (α/β)_OAR_. Since (α/β)_T_ < (α/β)_OAR_, escalating the total dose beyond the original 78 Gy level by adding fractions will increase the TCP/NTCP ratio less than by increasing the fraction dose. This becomes apparent from Table 1 when comparing the TCP and NTCP scores for the plans with a 5% nominally higher prescription dose of 82 Gy, obtained either from scaling the fraction size to 2.1 Gy (with 39 fractions) or from increasing the number of fractions to 41 (with 2 Gy per fraction). The opposite holds in case of dose de-escalation below 78 Gy and for (α/β)_T_ > (α/β)_OAR_. When plotted in the same graph, both TOC curves would coincide only if (α/β)_T_ = (α/β)_OAR_.

**Table 1:** Dose and response statistics for renormalised 3D-CRT plans

		**Dose-per-fraction (*N*** **= 39)**			**Number of fractions (2 Gy/fraction)**		
		**95%**	**100%**	**105%**	***N*** **= 37**	***N*** **= 39**	***N*** **= 41**
prostate							
	D_min_	73.2	77.1	80.9	73.1	77.1	81.1
	D_mean_	74.3	78.2	82.1	74.2	78.2	82.2
	D_max_	75.1	79.1	83.1	75.0	79.1	83.2
	TCP	76%	83%	89%	78%	83%	87%
rectum							
	D_mean_	45.3	47.7	50.1	45.3	47.7	50.1
	D_max_	74.9	78.9	82.8	74.9	78.9	82.9
	NTCP_GI_	13%	25%	44%	16%	25%	38%
bladder							
	D_mean_	31.6	33.3	34.9	31.6	33.3	35.0
	D_max_	74.0	77.9	81.8	73.9	77.9	81.9
	NTCP_GU_	13%	15%	16%	13%	15%	19%

*Abbreviations: N*, number of fractions; *D*_*min*_, minimum dose; *D*_*mean*_, mean dose; *D*_*max*_, maximum dose, *TCP*, tumour control probability, *NTCP*_*GI*_, probability of late gastrointestinal morbidity; *NTCP*_*GU*_, probability of late genitourinary morbidity.

**Figure 5:**
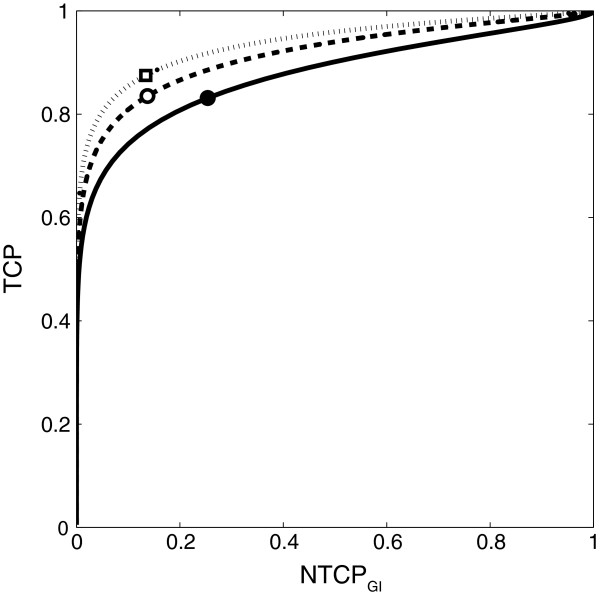
**TOC graphs for 3D-CRT, IMRT**_**phys **_**and IMRT**_**biol **_**plans.** TOC graphs for TCP *vs.* NTCP_GI_ obtained by dose level scaling of a prostate 3D-CRT plan (solid curve), the IMRT_phys_ plan (dashed curve), and the IMRT_biol_ plan (dotted curve) at constant fraction number, *N* = 39. Symbols (● , **○**, **□**) represent the TCP and NTCP scores of the initial plans.

In Figure 5, TOC graphs for the 3D-CRT, IMRT_phys_, and IMRT_biol_ plans for the same patient anatomy are shown. Dose statistics for the initial plans are given in Table 2. By comparing the TOC graphs for the 3D-CRT and the IMRT plans, it is obvious that the IMRT_phys_ plan has a more conformal dose distribution than the 3D-CRT plan, reducing NTCP (from 25% to 13%) at constant TCP = 83%. Comparison of the TOC graphs for the IMRT_phys_ and the IMRT_biol_ plan suggests that an increase in TCP (from 83% to 87%) can be obtained at constant NTCP =13%. It can be seen that the three TOC graphs do not cross and have different AUCs (3D-CRT: 0.87, IMRT_phys_: 0.91, IMRT_biol_: 0.93). The IMRT_biol_ plan outperforms the IMRT_phys_ plan over the whole range of prescribed dose levels and fractionation schemes, whereas the latter outperforms the 3D-CRT plan. By comparing the TOC graphs, its use for prescription dose customisation becomes apparent; when rescaling a given treatment plan does not fulfill the TCP/NTCP trade-off requirements, only re-optimization (with more direct steering of the TCP/NTCP criteria) will improve the quality of the plan.

**Table 2:** Dose and response statistics for initial treatment plans (39 fractions)

		**3D-CRT**	**IMRT**_**phys**_	**IMRT**_**biol**_
prostate				
	D_min_	77.1	75.4	74.1
	D_mean_	78.2	78.8	81.8
	D_max_	79.1	81.8	97.0
	TCP	83%	83%	87%
rectum				
	D_mean_	47.7	36.8	36.3
	D_max_	78.9	80.1	84.7
	NTCP_GI_	25%	13%	13%
bladder				
	D_mean_	33.3	25.1	17.7
	D_max_	77.9	82.4	93.8
	NTCP_GU_	15%	14%	14%

*Abbreviations: 3D-CRT*, three-dimensional conformal radiation therapy; *IMRT*_*phys*_, physically optimised IMRT plan; *IMRT*_*biol*_, physico-biologically optimised IMRT plan; *D*_*min*_, minimum dose; *D*_*mean*_, mean dose; *D*_*max*_, maximum dose, *TCP*, tumour control probability, *NTCP*_*GI*_, probability of late gastrointestinal morbidity; *NTCP*_*GU*_, probability of late genitourinary morbidity.

Figure 5 clearly shows that both the TOC’s shape and its AUC help to estimate the therapeutic power of a treatment plan over a continuous range of TCP and NTCP scores. Finally, the TOC graph of the best performing (IMRT_biol_) plan can be presented to the physician and/or patient to assess the trade-off between treatment benefit and morbidity and to choose from the TOC graph the dose-level that best fits their preferences.

## Discussion

In an era of individualised cancer therapy, radiotherapy should move towards customised dose prescription in order to maximise individual patient’s outcome and quality of life. The currently applied ‘rigid’ treatment protocols neither take into account the anatomical diversity of patients within the risk group nor their individual risk-taking preferences. The standard strategy will lead to relative underdosage in individuals who are willing to tolerate a higher radiation dose aiming for tumour control, whilst relatively overdosing those not willing to accept the possible adverse effects resulting from the predefined radiation dose. Hence, there is a need to move toward customised treatment planning where individualisation is not restricted to adapting the spatial dose distribution to the patient’s anatomy, but also involves balancing of treatment benefit and morbidity in terms of TCP and NTCP.

As an overture towards individualised TCP and NTCP risk balancing, we conducted a prospective decision-making trial in patients with localised prostate carcinoma scheduled for 3D-CRT, who were offered an *a priori* restricted choice between treatments with two alternative pre-selected dose levels [2]. To take this one step further, a tool to depict a continuum of clinically relevant dose levels and their corresponding TCP and NTCP indices would be helpful to assist selecting an optimum dose level after an initial treatment plan has been generated for the individual patient.

In previous work by Lind *et al.*, the concept of maximizing the probability of uncomplicated tumour control, *P*_+_, as a function of dose has been proposed to find the optimum dose level [10]. It should be noted that the TOC graph provides unbiased information in comparison to the *P*_+_ graph when plotted as a function of dose. This becomes clear when the general expression for *P*_+_ is considered:

(1) $$P_{+}=\left(\text{TCP}-\text{NTCP}+\delta \left\{\text{NTCP}\left(1-\text{TCP}\right)\right\}\right),$$

where *δ* is the estimated fraction of patients for which tumour and normal tissue response are statistically independent (0 ≤ *δ* ≤ 1) [9]. Expression (1) assumes that an implicit *a priori* trade-off between TCP and NTCP is made. For example, with *δ* = 0 equation (1) reduces to *P*_*+*_ *= TCP – NTCP*, assuming that the risk of recurrence is equally important as the risk of suffering from (severe) side effects. Therefore, *P*_+_ has often been criticised as a measure that does not reflect the clinical reality that reductions in TCP are rated differently from the risk of complications by clinicians and patients. By plotting TCP *vs.* NTCP with the prescribed dose as an independent parameter, no assumption with regard to *a priori* risk-taking preferences between TCP and NTCP is made. Instead, their interrelationship is assessed over the whole range of potential dose levels to facilitate the selection of an optimum that satisfies the risk-taking preferences of the individual clinician or patient. Using the full TOC graph instead of selecting the single dose level where *P*_+_ is maximal pre-empts the criticism against *a priori* balancing the TCP/NTCP trade-off.

In the 1970’s Moore and Mendelsohn proposed the TOC curve as a method to optimise treatment levels in cancer therapy [11] and later on, it was used in studies on radiation therapy for head and neck tumours [12-14]. However, this concept has only been employed to determine an optimum dose level for a patient population in a ‘one size fits all’ approach. We emphasize that with the current possibilities and knowledge of inverse treatment planning techniques (*e.g.*, by explicitly incorporating dose-response relationships into the optimization as objective and/or constraint functions) the application options for individualized dose prescription strategies may be more important and clinically relevant than before.

The TOC graph used in the current work provides a means to visualise and explore the trade-off between TCP and NTCP in an intuitive manner to be used as a tool for *a posteriori* dose prescription customisation of an initial treatment plan for an individual patient. Plans with different TCP/NTCP trade-offs can be generated from the same underlying relative dose distribution by scaling of the prescribed dose or fractionation scheme. Since no re-planning is required, the TOC graph can be generated off-line. Furthermore, this approach facilitates the interactive balance between TCP and NTCP of a given plan *after* treatment planning and plan optimisation and does not require risk-taking predilections to be articulated *a priori*, as is the case in today’s inverse treatment planning approaches. Additionally, by exploiting the AUC, a quantitative definition of the therapeutic power is provided independent of consensus on the dose level.

The clinical application of changing the prescribed dose and fractionation after an initial treatment plan has been generated has recently gained renewed interest as part of individualised dose prescription strategies that escalate the tumour dose until maximally tolerable NTCP limits are reached in, for example, non-small cell lung cancer radiotherapy [6,7,21,22]. Current treatment planning systems lack the means to assess the effects of dose or fractionation variation of a given treatment plan in terms of TCP and NTCP indices and do not provide insight in their interrelationship. Consequently, it is common practice to completely re-design and re-calculate a treatment plan once the dose description or fractionation schedule has changed. Our re-scaling approach together with the TOC concept brings individualised dose prescription into clinical practice by providing an intuitive and easy-to-apply tool to find the preferred prescription dose for either a fixed number of fractions (by changing the dose per fraction) of for fixed fraction dose (by changing the number of fractions) which yields pre-selected NTCP limit(s) for the OAR(s).

### Practical use of TOC graph

To exploit our concept in the clinical workflow, first, a TOC graph is generated from historical dose-response data that were derived for a *group* of patients treated with the same irradiation technique but with different dose prescriptions. The *individual* patient and the radiation oncologist interactively choose TCP/NTCP coordinates from the TOC graph in a first decision-making step, which results in an *intended* dose prescription. Subsequently, an initial treatment plan is designed for the intended dose prescription based on the individual patient’s anatomy. Hence, the population-based TOC can be used to establish an evidence-based ‘customised’ dose prescription for a given treatment technique prior to designing a treatment plan for the *intended* dose prescription level. Finally, a second decision-making step is required to further customise the dose prescription by re-normalisation and to achieve at least the desired, but likely superior, TCP/NTCP scores.

### Physico-biological treatment plan optimisation

We showed that constrained optimisation in inverse planning based on TCP and NTCP models resulted in an iso-toxic treatment plan with improved TCP. As the TCP model allows for some degree of dose heterogeneity in the PTV, a dose reduction in parts of the PTV is exploited to reduce the NTCP, whereas in other parts the dose is escalated. A steep dose fall-off at the border of the PTV keeps the TCP constant, and allows for better sparing of the OAR, which facilitates dose escalation resulting in the same NTCP with higher TCP.

### Relation of TOC graph to Pareto efficient frontier

The TOC graph should not be confused with the Pareto efficient frontier (PEF) that has been discussed in radiotherapy lately (*e.g.*, [23]). The principles of TOC and PEF are fundamentally different. Whereas the TOC graph has been obtained from scaling a single treatment plan, a PEF would have required the physico-biological optimisation problem to be repeatedly solved to optimality for different *a priori* set NTCP constraint values. A comparison between the TOC and PEF is however beyond the scope of this paper and will be addressed in another paper by the authors.

Due to uncertainties in the radiobiological models, the choice for a final dose level should not solely be based on the TOC graph, but should always include the underlying 3D dose distribution. Preferably, the TOC graph should be presented together with the model parameters, TCP/NTCP values, and confidence levels [13].

However, confidence intervals for all model parameters are not available or may be unreliable for IMRT dose distributions, as they have mainly been derived from 3D conformal radiotherapy dose distributions. As more clinical data comes available the TOC-based estimate of the optimal dose level will get more trustworthy. We therefore recommend to carefully introduce the TOC into clinical practice, by gradually releasing the interval of dose-level re-scaling towards values where the uncertainties are largest. The true value of the TOC graph and underlying TCP/NTCP models should be assessed in clinical trials comparing the predicted and actual TCP and NTCP values.

## Conclusions

For an individual patient, the TOC graph can be exploited as an *a posteriori* decision aid in risk-adapted dose prescription customisation of a given treatment plan as a function of the prescribed dose level or the number of fractions. It provides physicians and patients with a decision aid for individual risk-taking preferences in terms of TCP/NTCP trade-off. The AUC is a dose level independent measure of the therapeutic power of a treatment plan.

## Abbreviations

AUC: Area under the curve; bNED5: 5-year genitourinary biochemical no evidence of disease; DVH: Dose-volume histogram; EQD2: Equivalent dose in 2 Gy fractions; GI: Gastrointestinal; GU: Genitourinary; ICRU: International commission on radiation units and measurements; IMRT: Intensity-modulated radiation therapy; IMRTphys: Physically optimised IMRT plan; IMRTbiol: Physico-biologically optimised IMRT plan; MV: Megavolt; NTCP: Normal tissue complication probability; OAR: Organ at risk; P+: Uncomplicated tumour control probability; PEF: Pareto efficient frontier; PTV: Planning target volume; ROC: Receiver operating characteristic; RTOG: Radiation therapy oncology group; TCP: Tumour control probability; TOC: Therapeutic operating characteristic; 3D-CRT: Three-dimensional conformal radiation therapy

## Competing interests

All authors declare to have no competing interests.

## Authors’ contributions

ALH, conceived and designed the study, carried out the data collection, performed the data analysis and interpretation, and drafted the manuscript. HH, participated in the design of the study and the interpretation of the data, and helped to draft the manuscript. JHK, contributed substantially to the critical revision of the paper. All authors read and approved the final manuscript.

## Supplementary Material

Additional file 1 TCP and NTCP models used in systematic literature review. Click here for file

Additional file 2 TCP and NTCP models used by ORBIT Workstation. Click here for file
