# Supplementary material for: Employing the therapeutic operating characteristic (TOC) graph for individualised dose prescription
Source: Radiat Oncol. 2013 Mar 7;8:55. doi: 10.1186/1748-717X-8-55 (PMC3606307; doi:10.1186/1748-717X-8-55)
Supplement: Additional file 1 — TCP and NTCP models used in systematic literature review. [file 1748-717X-8-55-S1.docx]

**Additional file 1**

**TCP and NTCP models used in systematic literature review**

In [3], a Probit model has been used to describe TCP as a function of the prescribed dose level:

$TCP=\Phi\left(\frac{D-TD_{50}}{m\cdot TD_{50}}\right)$ , (I.1)

where

$\Phi(z)=\frac{1}{\sqrt{2\pi}}\int_{-\infty}^{z} \exp(-t^{2}/2)dt$ (I.2)

is the standard normal cumulative distribution function, *D* is the prescribed equivalent total dose in 2 Gy fractions, *TD*_50_ is the effective dose at the 50% response level and *m* determines the slope of the sigmoidal function $\Phi$. The model parameters used are *TD*_50_ = 56.18 Gy and *m* = 0.39 for the overall bNED. These values were derived from a fit over the dose domain of 60–80 Gy in 2 Gy fractions.

The NTCP model used in [3] is a logistic regression model:

$NTCP=\frac{1}{1+e^{-a-bD}}$ , (I.3)

where *D* is the prescribed equivalent total dose in 2 Gy fractions, and *a* and *b* are fitting parameters. Parameter values *a* = -11.08 and *b* = 0.06 gave the best fit over the relevant dose domain for gastrointestinal toxicity, while for genitourinary toxicity parameter values *a* = -6.47 and *b* = 0.03 resulted from the fitting procedure.
