# Supplementary material for: Employing the therapeutic operating characteristic (TOC) graph for individualised dose prescription
Source: Radiat Oncol. 2013 Mar 7;8:55. doi: 10.1186/1748-717X-8-55 (PMC3606307; doi:10.1186/1748-717X-8-55)
Supplement: Additional file 2 — TCP and NTCP models used by ORBIT Workstation. [file 1748-717X-8-55-S2.docx]

**Additional file 2**

**TCP and NTCP models used by ORBIT Workstation**

The linear-quadratic Poisson model was used to calculate the TCP for a heterogeneous dose distribution [7]:

$TCP=\prod_{i} \left(\exp(-\exp(e\cdot\gamma_{37}-(e\cdot\gamma_{37}-\ln(\ln2))\frac{EQD_{2}(D_{i})}{TD_{50}}\right)^{v_{i}}$ , (II.1)

where *TD*_50_ is the dose at the 50% response level, $\gamma_{37}$is the normalized dose-response gradient at the 37% response level, and *D*_i_ and *v*_i_ are the dose bins and relative volumes of the dose-volume histogram (DVH), respectively. The model parameters *TD*_50_ = 56.18 Gy and $\gamma_{37}$ = 1.3 and α/β = 2 Gy perfectly match the dose-response of the Probit TCP model (I.1) over the dose domain of 60–80 Gy in 2 Gy fractions. For NTCP calculation, the Lyman-Kutcher-Burman model was used with 2 Gy fractionation correction based on the linear-quadratic cell survival model [23,24]:

$NTCP=\Phi\left(\frac{gEQD_{2,a}-TD_{50}}{m\cdot TD_{50}}\right)$ , (II.2)

where $\Phi(z)$ is the standard normal cumulative distribution function according to (I.2), and

$gEQD_{2,a}=\left[\sum_{i} v_{i}\cdot EQD_{2}(D_{i})^{a}\right]^{1/a}$ , (II.3)

is the generalized uniform dose for a 2 Gy fractionation corrected DVH with dose bins *D*_i_ and relative volume *v*_i_. Model parameters for Grade ≥ 2 late rectal toxicity (*TD*_50_ = 76.9 Gy, *m* = 0.13, *a* = 12.5) and α/β = 3 Gy were adopted from [16]. Model parameters for Grade ≥ 2 late bladder toxicity (*TD*_50_ =62 Gy, *m* = 0.11, *a* = 7.7) and α/β = 6 Gy were adopted from [17].
